# Lethal Effects of *Aspergillus niger* against Mosquitoes Vector of Filaria, Malaria, and Dengue: A Liquid Mycoadulticide

**DOI:** 10.1100/2012/603984

**Published:** 2012-05-01

**Authors:** Gavendra Singh, Soam Prakash

**Affiliations:** Environmental and Advanced Parasitology and Vector Control Biotechnology Laboratories, Department of Zoology, Faculty of Science, Dayalbagh Educational Institute, Dayalbagh, Agra 282005, India

## Abstract

*Aspergillus niger* is a fungus of the genus *Aspergillus*. It has caused a disease called black mold on certain fruits and vegetables. The culture filtrates released from the *A. niger* ATCC 66566 were grown in Czapek dox broth (CDB) then filtered with flash chromatograph and were used for the bioassay after a growth of thirty days. The result demonstrated these mortalities with LC_50_, LC_90_, and LC_99_ values of *Culex quinquefasciatus* 0.76, 3.06, and 4.75, *Anopheles stephensi* 1.43, 3.2, and 3.86, and *Aedes aegypti* 1.43, 2.2, and 4.1 **μ**l/cm^2^, after exposure of seven hours. We have calculated significant LT_90_ values of *Cx. quinquefasciatus* 4.5, *An. stephensi* 3.54, and *Ae. aegypti* 6.0 hrs, respectively. This liquid spray of fungal culture isolate of *A. niger* can reduce malaria, dengue, and filarial transmission. These results significantly support broadening the current vector control paradigm beyond chemical adulticides.

## 1. Introduction

Rapidly emerging insecticide resistance is creating an urgent need for new active ingredients to control the adult mosquitoes [1]. A range of isolates belonging to the fungal species *Metarhizium anisopliae *and *Beauveria bassiana* have been shown to infect and significantly reduce the longevity of adult *Anopheles *mosquitoes, killing them within 14 days [2–4]. The *Beauveria bassiana* has infected mosquitoes of the insecticide resistant *Anopheles arabiensis* at two different temperatures [5]. The fungi have been applied by spraying on mosquitoes with an oil formulation of infectious spores. The fungal spores begin pathogenic and invade the mosquitoes, after which the fungus multiplies and kills its host within two weeks [2]. Similarly, the isolates of *Metarhizium anisopliae *ICIPE-30 and *Beauveria bassiana *I93-825 (IMI 391510) have reduced mosquito survival on immediate exposure and up to 28 days after application [6]. The critics have argued that “slow acting” these fungal biopesticides is, therefore, incapable of delivering mosquito control in different parts of the world. The entomopathogenic fungi can be integrated into control programmes additional information regarding isolate selection, optimisation of production, and formulation is required. While many successful laboratory evaluations of the efficacy of entomopathogenic fungi have been conducted [2, 7–9]. Therefore, more research on fungal formulations and evaluating of various formulations, delivery techniques remains essential against mosquitoes.

*Aspergillus niger* is a filamentous ascomycete fungus that is ubiquitous in the environment and has been implicated in opportunistic infections of humans [10]. *A*. *niger* is most widely known for its role as a citric acid producer [11]. With production of citric acid at over one million metric tons annually, *A*. *niger* citric acid production serves as a model fungal fermentation process. This organism is a soil saprobe with a wide array of hydrolytic and oxidative enzymes involved in the breakdown of plant lignocellulose. A variety of these enzymes from *A*. *niger* is important in the biotechnology industry. The *A*. *niger* is also an important model organism for several important research areas including the study of eukaryotic protein secretion in general, the effects of various environmental factors on suppressing or triggering the export of various biomass degrading enzymes, molecular mechanisms critical to fermentation process development, and mechanisms involved in the control of fungal morphology. These are encouraging characteristics which encourage for further research on mosquitoes control.

 Mosquito vectors are solely responsible for transmitting diseases, such as malaria, dengue, chikungunya, Japanese encephalitis, yellow fever, and lymphatic filariasis [12]. Malaria is an important cause of death and illness in children and adults, especially in tropical countries. Malaria is caused by a parasite that is transmitted from one human to another by the bite of infected *Anopheles stephensi*. Half of the world's population is at risk from malaria. Each year almost 250 million cases occur, causing 860000 deaths [13]. Approximately 3.5 billion people live in dengue endemic countries which are located in the tropical and subtropical regions of the world [14]. Lymphatic filariasis, commonly known as elephantiasis, is so far a neglected tropical disease. The infection occurs when filarial parasites are transmitted to humans through *Culex quinquefasciatus*. More than 1.3 billion people in eighty-one countries worldwide are threatened by lymphatic filariasis [15]. In the present investigation, we have reported the lethal effects of purified culture filtrates of *A*. *niger* against *An*. *stephensi*, *Cx*. *quinquefasciatus*, and *Ae*. *aegypti* in the laboratory.

## 2. Materials and Methods

### 2.1. Collection and Culture of *Aspergillus niger*

**Figure 1:**
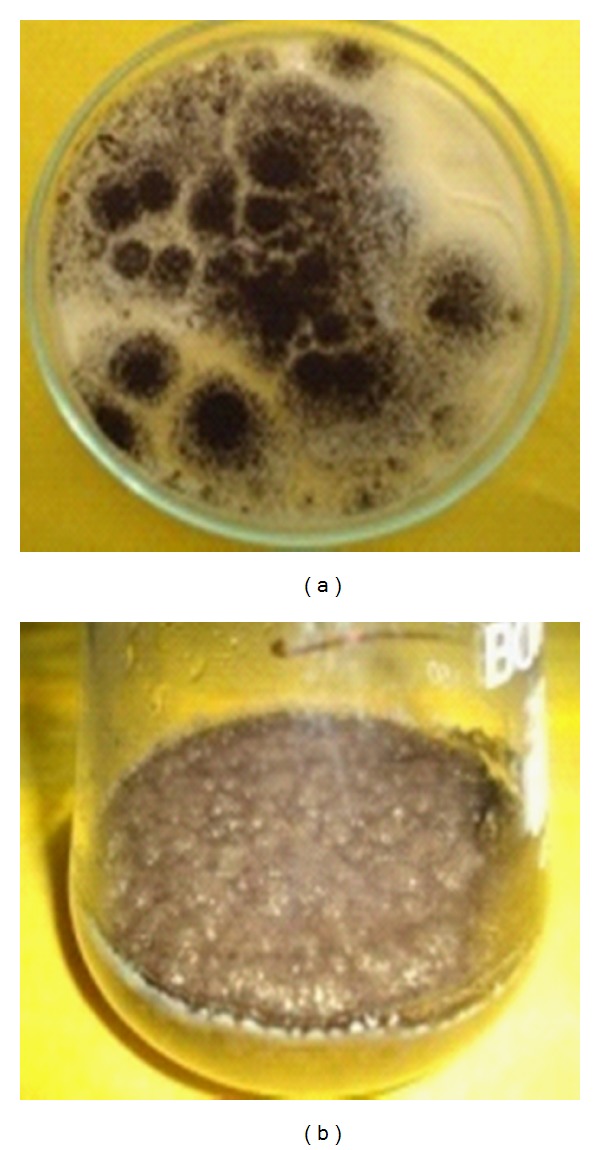
The cultures of *Aspergillus niger*: (a) solid medium on Czapek dox agar (CDA), (b) liquid medium Czapek dox broth maintained in the laboratory.

The strain of *Aspergillus niger* (ATCC 66565) was obtained from Microbial Type Culture Collection and Gene Bank (MTCC 2587) Institute of Microbial Technology, Chandigarh, India. *A*. *niger* was maintained on autoclaved Czapek dox broth (sucrose: 30.0 g, sodium nitrate: 3.0 g, dipotassium phosphate: 1.0 g, magnesium sulphate: 0.05 g, potassium chloride: 0.05 g, ferrous sulphate: 0.01 g, deionized water: 1000 mL) and adjusts pH 6.5. The broth was supplemented with 50 *μ*g/mL chloramphenicol as a bacteriostatic agent. The colonies of *A*.* niger* were grown on Czapek dox agar (CDA), solid medium plates were transferred to each flask using an inoculation needle. The conical flasks, inoculated with *A*.* niger*, were incubated at 25°C for 30 days (Figure 1).

### 2.2. Preparation of Flash Chromatograph Columns and Filtration

In the Flash chromatograph, a plastic column was filled with silica gel, with the sample to be separated placed on top of this support. The rest of the column is filled with an isocratic or gradient solvent which, with the help of pressure, enables the sample to run through the column and become separated. Flash chromatography used air pressure initially to speed up the separation. The culture filtrates were obtained by filtering the broth through Whatman no.1 filter paper. These metabolites were further filtered with the flash chromatograph.

### 2.3. Bioassays

The flash chromatograph purified culture filtrates were used for bioassays against laboratory reared *Cx*. *quinquefasciatus*, *Ae. aegypti*, and *An. stephensi* as per the standard procedures recommended by World Health Organization with some modifications [16]. The freshly emerged three-day-old sugar fed adults were used for the assay. The five different volumes of 1.6, 2.2, 2.7, 3.2, and 3.8 *μ*L/cm^2^ of metabolites were sprayed in a cage (25 cm length × 15 cm width × 5 cm depth) containing 25 mosquitoes. The exposed mosquitoes were kept under observation, and dead mosquitoes were discarded daily. Each bioassay including control was conducted in triplicate on different days. In the control cages deionized water was sprayed. Daily mortality counts were performs. The bioassays were carried out at room temperature with 75 ± 5% relative humidity. The negative control was deionized water with 1% CDB while the positive control was Gokilaht-S 5EC (*d,d-trans*-cyphenothrin).

### 2.4. Statistical Analysis

The efficacy study of the filtrate metabolites of* A*.* niger* was assessed against *Cx*. *quinquefasciatus*, *Ae*. *aegypti*, and *An*. *stephensi *by probit analysis [17] with the statistical package IBM SPSS 19.0.

## 3. Results and Discussion

In the present observations, we have evaluated the lethal effects of culture filtrates of *A*. *niger* against adult mosquitoes. The lethal effects of *A*. *niger* with LC_50_, LC_90_, and LC_99_ values of *Cx*.* quinquefasciatus* were 0.76, 3.06, and 4.751 *μ*L/cm^2^. Moreover, in case of the *An*.* stephensi* it was observed as 1.43, 3.2, and 3.86. While in case of *Ae*.* aegypti* it was recorded as 1.43, 2.2, and 4.1 *μ*L/cm^2^. These values were calculated after the exposure of seven hours along with their probit quotations (Table 1). The entomopathogenic fungus has been successfully reduceing mosquito vectors population in laboratories and field trials [2–4, 18]. The fungal infections for the mosquitoes become increasingly sick and are eventually killed, but the process can take up to a week or more. The adult mosquitoes pick up the fungal spores when resting on treated surfaces.

 Unlike fast-acting chemical neurotoxins, fungal pathogens do not cause rapid mortality or immediate “knockdown” but rather act over a number of days as the fungal spores penetrate the insect cuticle and then proliferate within the hemocoel [19]. The *A. clavatus *has been found highly pathogenic against larvae of *Ae. aegypti*, *Cx. quinquefasciatus*, and *An. gambiae* [20]. The mortality rates were 100% against both *Ae. aegypti*, and *Cx. quinquefasciatus*, while against *An. gambiae* it was 95% after 24 hours. The entomopathogenic fungus *B*. *bassiana* has used as an alternative vector control tool against insecticide-resistant mosquitoes under conditions typical of indoor resting environments [5]. A range of fungal-based insecticide combinations was used to test effects of timing and sequence of exposure. Both the laboratory-reared and field-collected mosquitoes were highly resistant to permethrin but susceptible to *B*. *bassiana* and *M*. *anisopliae* infection, inducing 100% mortality within nine days. Simultaneous coexposure induced the highest mortality, up to 70.362% for a combined *Beauveria* and permethrin exposure within a time range of one gonotrophic cycle (4 days) [21]. In present investigation the lethal time effect of *A*. *niger* with LT_50_ and LT_90_ values of *Cx*. *quinquefasciatus* 2.57, 4.5, *An*. *stephensi* 1.58, 3.54, and *Ae*. *aegypti* 1.65, 6.0 hrs were calculated (Table 1). At the first time for increase in percent mortalities, a combination of an insecticide and an entomopathogenic fungus has been tested against *Ae*. *aegypti*. It can be an alternative to applications of high concentrations of chemical insecticides. The *Ae*. *aegypti* could be controlled by surface application of entomopathogenic fungi and that the efficiency of these fungi increased by combining the fungi with ultra-low concentrations of insecticides, resulting in higher mortality following relatively short exposure times [22].

**Figure 2:**
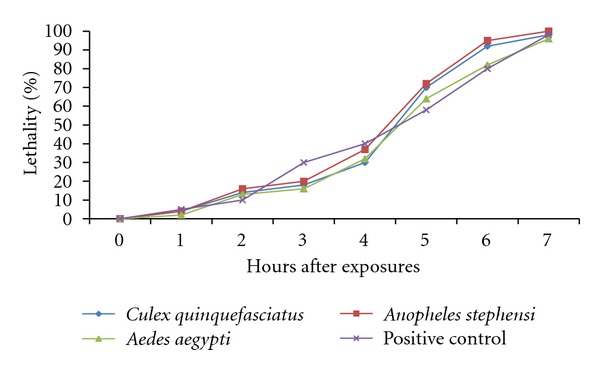
Effect of culture filtrates of *Aspergillus niger* against *Culex quinquefasciatus*, *Anopheles stephensi*, and *Aedes aegypti* after exposure of 7 hours.

**Figure 3:**
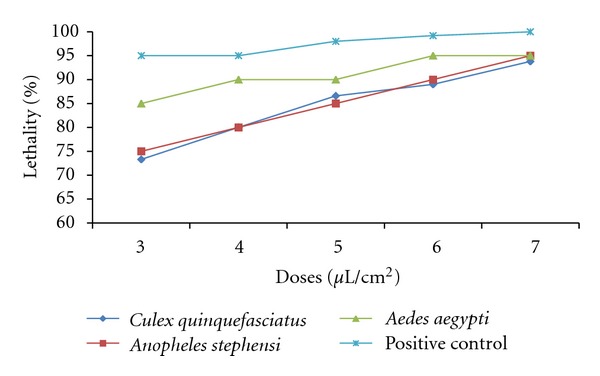
Effect of culture filtrates of *Aspergillus niger* against *Culex quinquefasciatus*, *Anopheles stephensi* and *Aedes aegypti* at different concentrations.

 This study distinctly demonstrates that the *A*.* niger* culture filtrates have induced a higher impact on adult mosquitoes with significant percent of mortalities (Figure 2). The applied concentrations have affected *Cx*. *quinquefasciatus*, *An*. *stephensi*, and *Ae*. *aegypti* with relevant LC_50_, LC_90_ and LC_99_ values after exposure of seven hours (Figure 3). The recorded lethal effects show the potential for integrated fungus control measures to dramatically reduce malaria, filarial, and dengue vectors. The pathogenic fungi produce a wide variety of toxic metabolites, which vary from low molecular weight products of secondary metabolism to complex cyclic peptides and proteolytic enzymes [23]. A significant progress has been made in understanding enzymes involved with the penetration of host cuticle and the role of mosquitocidal toxins. The fungal metabolites can be more effective by joint action of numerous toxins and enzymes.

 The *A*. *niger* is the best producer of extracellular lipase [24]. The present study shows that the *A*. *niger* purified fungal culture filtrates have enhanced their lethal effects against *An*. *stephensi*, *Cx*. *quinquefasciatus*, and *Ae*. *aegypti*. Moreover, the presence of mycotoxin “ochratoxin” in *A*. *niger* can be fast-acting metabolites for control of adult mosquitoes. Ideally, all these new findings could be implemented with a time application with its fast acting impact against *An*. *stephensi*, *Cx*. *quinquefasciatus*, and *Ae*. *aegypti* populations. This investigation can be further improved by implementing enhanced fungus-based strategy to control the adult population. In our laboratories, *Trichophyton ajelloi*, *Chrysosporium lobatum*, *C*. *tropicum*, *Lagenidium giganteum*, *Culicinomyces clavisporus*, and *Fusarium oxysporum* have so far been successfully screened and were found pathogenic against larvae and adults of *An*. *stephensi*, *Cx*. *quinquefasciatus*, and *Ae*. *aegypti*. These fungal strains have shown lethal effect after exposure of 24, 42, and 72 hours. Consequently, *A*. *niger* has found effective in very short time. This unique property of this fungus requires further field testing in different climatic zones.

## Figures and Tables

**Table 1:** Adulticidal activities of culture filtrates of *Aspergillus niger* against *Culex quinquefasciatus* (Say), *Anopheles stephensi* (Liston), and *Aedes aegypti* (Lin.).

Concentrations	Lethality (%)				LC_50_	LC_90_	LC_99_	LT_50_	LT_90_
(*μ*L/cm^2^)	*Culex quinquefasciatus*	*Anopheles stephensi*	*Aedes aegypti*		(*μ*L/cm^2^)^c^	(*μ*L/cm^2^)^c^	(*μ*L/cm^2^)^c^	(hrs)^d^	(hrs)^d^
0.0	0.0	0.0	0.0	*Culex quinquefasciatus *	0.76	3.06	4.75	2.57	4.5
1.6	73.3	75	85	*y* = 0.81 + 8.58*x*	(0.11–1.36)	(2.48–3.64)	(4.15–5.49)	(1.32–3.82)	(3.29–5.85)
2.2	80	80	90	*Anopheles stephensi *	1.43	3.2	3.86	1.58	3.54
2.7	86.6	85	90	*y* = 0.14 + 8.82*x*	(0.86–2.0)	(2.62–3.77)	(3.29–4.33)	(0.41–2.75)	(2.37–4.41)
3.2	89	90	95	*Aedes aegypti*	1.43	2.2	4.1	1.65	6.0
3.8	93.8	95	95	*y* = 0.19 + 9.23*x*	(0.816–1.92)	(1.77–2.93)	(3.8–4.91)	(0.61–2.69)	(4.96–7.04)
Gokilaht-S 5EC^a^	100	100	100						
Deionized water +CD^b^	00	00	00						

^a^Positive control.

^b^Negative control.

^c^Lethal concentrations with the corresponding 95% confidence level.

^d^Lethal time with the corresponding 95% confidence level.
